# Feature Selection for Better Identification of Subtypes of Guillain-Barré Syndrome

**DOI:** 10.1155/2014/432109

**Published:** 2014-09-15

**Authors:** José Hernández-Torruco, Juana Canul-Reich, Juan Frausto-Solís, Juan José Méndez-Castillo

**Affiliations:** ^1^División Académica de Informática y Sistemas, Universidad Juárez Autónoma de Tabasco, Km. 1 Carretera Cunduacán-Jalpa de Méndez, Colonia La Esmeralda, 86690 Cunduacán, TAB, Mexico; ^2^Universidad Politécnica del Estado de Morelos, Boulevard Cuauhnáhuac 566, Colonia Lomas del Texcal, 62574 Jiutepec, MOR, Mexico; ^3^Hospital General de Especialidades “Dr. Javier Buenfil Osorio”, Avenida Lázaro Cárdenas 208, Colonia Las Flores, 24097 San Francisco de Campeche, CAM, Mexico

## Abstract

Guillain-Barré syndrome (GBS) is a neurological disorder which has not been explored using clustering algorithms. Clustering algorithms perform more efficiently when they work only with relevant features. In this work, we applied correlation-based feature selection (CFS), chi-squared, information gain, symmetrical uncertainty, and consistency filter methods to select the most relevant features from a 156-feature real dataset. This dataset contains clinical, serological, and nerve conduction tests data obtained from GBS patients. The most relevant feature subsets, determined with each filter method, were used to identify four subtypes of GBS present in the dataset. We used partitions around medoids (PAM) clustering algorithm to form four clusters, corresponding to the GBS subtypes. We applied the purity of each cluster as evaluation measure. After experimentation, symmetrical uncertainty and information gain determined a feature subset of seven variables. These variables conformed as a dataset were used as input to PAM and reached a purity of 0.7984. This result leads to a first characterization of this syndrome using computational techniques.

## 1. Introduction

Guillain-Barré syndrome (GBS) is an autoimmune neurological disorder characterized by a fast evolution, generally from a few days up to four weeks [1]. GBS has an incidence of 1.3 to 2 per 100,000 people and a mortality rate from five to fifteen percent. The exact cause of GBS is unknown; however, it is frequently preceded by either a respiratory or a gastrointestinal infection. The diagnosis of GBS includes clinical, serological, and electrophysiological criteria [2]. The severity of GBS varies among subtypes, which can be mainly acute inflammatory demyelinating polyneuropathy (AIDP), acute motor axonal neuropathy (AMAN), acute motor sensory axonal neuropathy (AMSAN), and Miller-Fisher syndrome [1]. Electrodiagnostic criteria for distinguishing AIDP, AMAN, and AMSAN are well established in the literature [3], while the Miller-Fisher subtype is characterized by the clinical triad: ophthalmoplegia, ataxia, and areflexia [1].

A better understanding of the differences in the GBS subtypes is critical for the implementation of appropriate treatments for total recovery and in certain cases for the survival of patients. Hospitalization time and the cost of treatments vary according to the severity of the specific subtype. Finding a minimum feature subset to accurately identify GBS subtypes could lead to a simplified and cheaper process of diagnosis and treatment of the GBS case. The ultimate goal of a physician is to get patients to a full recovery. This can be more effectively achieved when an early diagnosis of the case is performed using a minimum number of medical features.

This work constitutes a first attempt to using machine learning techniques, specifically cluster analysis in combination with filter methods for feature selection. We aim at finding a small feature subset to identify four GBS subtypes. Machine learning techniques have been found in the literature to predict the prognoses of this syndrome [4, 5] as well as to find predictors of respiratory failure and necessity of mechanical ventilation in GBS patients [6–8]. Nevertheless, no previous publications about specific subtypes identification of the syndrome using machine learning techniques were found in the literature.

Cluster analysis is a computational technique from the machine learning area that is shown to be useful to find different groups of objects in datasets [9–12]. However, datasets might contain a mixture of “bad” and “good” features. “Bad” features are redundant or noisy features and make algorithms slow and inaccurate. Feature selection techniques allow reducing the dimensionality of a dataset such that it only contains “good” features which would maximize the performance of the algorithms and thus enabling the possibility of reaching a higher accuracy [13]. For feature selection, several machine learning methods are available, which are usually classified as filter [14–19], wrapper [20–22], embedded [23–25], and hybrid [26–29]. From the machine learning point of view it is interesting to analyze the performance of feature selection methods in diverse scenarios with real data, as this case is.

In this work we use a real dataset consisting of 156 features and 129 cases of GBS patients; these are 20 AIDP cases, 37 AMAN, 59 AMSAN, and 13 Miller-Fisher cases. The dataset contains clinical, serological, and nerve conduction tests data.

We use PAM (Partitions Around Medoids) clustering algorithm to identify with the highest purity groups corresponding to four subtypes of GBS. A group with high purity contains the largest number of elements of the same type and the fewest number of elements of a different type. Purity is an external clustering validation metric that evaluates the quality of a clustering based on the grouping of objects into clusters and comparing this grouping with the ground truth. Although there are several clustering validation metrics, both internal and external [30], we selected purity since our interest was to find “pure” groups and to take advantage of the available prior knowledge of the true labels. The use of a prior knowledge to evaluate a clustering process is also known as supervised or semisupervised clustering; some examples can be found in [31–34].

In order to achieve the identification of the four groups with a high purity it is necessary to select the relevant features in the dataset; otherwise the purity magnitude would be compromised as stated in [13]. For this initial exploratory study, we chose filter methods as they are the simplest and lowest computational demanding methods available in the literature and as they work independently of the clustering algorithms. We focus on five filter methods: correlation-based feature selection (CFS), chi-squared, information gain, consistency, and symmetrical uncertainty methods.

The experimental results showed a good performance of the method and allowed us to obtain a first characterization of GBS using machine learning techniques. 

## 2. Materials and Methods

### 2.1. Data

The dataset used in this work comprises 129 cases of patients seen at Instituto Nacional de Neurología y Neurocirugía located in Mexico City. Data were collected from 1993 through 2002. There are 20 AIDP cases, 37 AMAN, 59 AMSAN, and 13 Miller-Fisher cases. The identification of subtypes was made by a group of neurophysiologists based on the clinical and electrophysiological criteria established in the literature [1–3]. This dataset is not yet publicly available and this is the first time it is used in an experimental study. No public dataset was found to be used as a benchmark.

Originally, the dataset consisted of 365 attributes corresponding to epidemiological data, clinical data, results from two nerve conduction tests, and results from two cerebrospinal fluid (CSF) analyses. The second nerve conduction test was conducted in 22 patients and the second CSF analysis was conducted in 47 patients only. Therefore, data from these two tests were excluded from the dataset.

The diagnostic criteria for GBS are established in the literature [1–3]. These formal criteria were considered to determine which variables from the original dataset could be important in the characterization of the four subtypes of GBS. We made a preselection of variables based on these criteria. Originally, the dataset had 365 variables. After preselection, it was left with 156 variables: 121 variables from the nerve conduction test, 4 variables from the CSF analysis, and 31 clinical variables. As for the type of attributes, these are 28 categorical and 128 numeric attributes. The situation of dealing with mixed data types was solved using Gower's similarity coefficient, as explained later.

### 2.2. Filter Methods

We selected filter methods for this initial exploratory study as they are in computational terms the fastest and simplest methods available in the literature for feature selection. Filters work independently from any clustering algorithm and base their decision solely on characteristics of data.

We chose these five particular methods based on their performance reported in the literature [15, 17, 35, 36]. Chosen filters apply diverse criteria to evaluate feature relevance. Filters investigated are CFS, chi-squared, information gain, symmetrical uncertainty, and consistency.

#### 2.2.1. Correlation-Based Feature Selection (CFS)

CFS [14] evaluates two aspects of a feature subset: its capacity to predict the class and the correlation between the features of the subset. This method seeks to maximize the first aspect and minimize the second one. This method results in a feature subset with the highest capacity to predict the class and the least correlation between features of the subset. Given a feature subset *S* containing *k* features, CFS finds the goodness of *S* denoted (*M*_*S*_) as follows:

(1) $$\begin{array}{c} M_{S}=\frac{k\overset{-}{r_{cf}}}{\sqrt{k+k\left(k-1\right)\overset{-}{r_{ff}}}}, \end{array}$$

where $\overset{-}{r_{ff}}$ is the average correlation of all feature-feature pairs, and $k\overset{-}{r_{cf}}$ is the average correlation of all feature-class pairs.

#### 2.2.2. Chi-Squared

This method evaluates the chi-square statistic of each feature taken individually with respect to the class [15] and provides a feature ranking as a result. The chi-square test for a feature*f* and the class *c* is defined as follows:

(2) $$\begin{array}{c} X^{2}\left(f,c\right)=\frac{{N\left[P\left(f,c\right)P\left(\overset{-}{f},\overset{-}{c}\right)-P\left(f,\overset{-}{c}\right)P\left(\overset{-}{f},c\right)\right]}^{2}}{P\left(f\right)P\left(\overset{-}{f}\right)P\left(c\right)P\left(\overset{-}{c}\right)}, \end{array}$$

where *N* is the number of observations in the dataset, *P*(*x*, *y*) is the joint probability of *x* and *y*, and *P*(*x*) is the marginal probability of *x*.

#### 2.2.3. Information Gain

Information gain measures the goodness of a feature to predict the class given that the presence or absence of the feature in the dataset is known. This method delivers a ranking according to the goodness of each feature.

Information gain [16] of a feature *f*_*k*_ and a class *c*_*i*_ is defined as follows:

(3) $$\begin{array}{c} \text{IG}\left(f_{k},c_{i}\right)=\underset{c\in \left\{c_{i},{\overset{-}{c}}_{i}\right\}}{\sum }\;\underset{f\in \left\{f_{k},{\overset{-}{f}}_{k}\right\}}{\sum }P\left(f,c\right)\log_{2}\frac{P\left(f,c\right)}{P\left(f\right)P\left(c\right)}, \end{array}$$

where $c\in \left\{c_{i},{\overset{-}{c}}_{i}\right\}$ is the set of all classes, $f\in \left\{f_{k},{\overset{-}{f}}_{k}\right\}$ is the set of all features, *P*(*f*, *c*) is the joint probability of feature *f* and class *c*, and *P*(*f*) and *P*(*c*) are the marginal probabilities of *f* and *c*, respectively.

#### 2.2.4. Consistency

This method finds the smallest feature subset that presumably improves the discriminatory power of the original feature subset. This subset has the highest consistency. The consistency for a given feature subset *S* is computed as follows [17]:

(4) $$\begin{array}{c} \text{Consistency}=1-\text{inconsistency}\;\text{rate}. \end{array}$$

Let us define a pattern as a set of values for *S*. An inconsistency arises when two patterns match exactly all attributes except for the class. The inconsistency count for a pattern is the number of times it appears in the dataset minus the number of times it appears in the majority class. The inconsistency rate is the sum of all the inconsistency counts for all possible patterns of *S* divided by the total number of patterns [18].

#### 2.2.5. Symmetrical Uncertainty

This method measures the correlation between pairs of attributes using normalization of information gain. The normalization is performed to compensate for the bias of information gain to benefit attributes with more values and to ensure that they are comparable [17]. This method results in a feature ranking.

Symmetrical uncertainty is computed as follows [19]:

(5) $$\begin{array}{c} U\left(A,B\right)=2*\frac{MI\left(A,B\right)}{\text{Entropy}\;\left(A\right)+\text{Entropy}\;\left(B\right)}\\ MI\left(A,B\right)=\sum P\left(A,B\right)\log_{2}\frac{P\left(A,B\right)}{P\left(A\right)P\left(B\right)}, \end{array}$$

where *P*(*X*) is the marginal probability of feature *X*, *R*_*A*_ is the range of feature *A*, and *P*(*A*, *B*) is the joint probability of features *A* and *B*. Entropy is computed using the classical equation discussed in [17].

### 2.3. Clustering Algorithm: Partitions Around Medoids (PAM)

As stated before, the dataset used in this work combines categorical and numeric data. PAM is a clustering algorithm capable of handling such situations. It receives a distance matrix between observations as input. The distance matrix was computed using Gower's coefficient, explained later.

PAM, introduced by Kaufman and Rousseeuw [37], aims to group data around the most central item of each group, known as medoid, which has the minimum sum of dissimilarities with respect to all data points. PAM forms clusters that minimize the total cost *E* of the configuration, defined as

(6) $$\begin{array}{c} E=\overset{k}{\underset{i=1}{\sum }}\;\underset{o\in C_{i}}{\sum }\mathrm{dist}\left(o,m\right), \end{array}$$

where *k* is the number of clusters, *o* ∈ *C*_*i*_ is the set of objects in cluster *c*_*i*_, and dist⁡(*o*, *m*) is the distance between an object *o* and a medoid *m*.

PAM works as shown in Algorithm 1 [38].

### 2.4. Gower's Similarity Coefficient

Distance metrics are used in clustering tasks to compute the distance between objects. The distance computed is used by clustering algorithms to determine how much similar or dissimilar the objects are and what cluster they belong to. There are many distance metrics. Some of them deal with numeric data, like Euclidean, Manhattan, and Minkowski [38]. To deal with binary data the Jaccard coefficient and Hamming are often used [38]. For categorical data, some distance metrics are Overlap, Goodall, and Gambaryan [39].

In this work we used for experimentation a dataset that contains mixed data, that is, both categorical and numeric data. To deal with this situation we selected Gower's coefficient. It is a robust and widely used distance metric for mixed data. We used this coefficient to obtain a matrix of distances between observations as PAM requires. It was introduced by Gower in 1971 [40]. Gower's coefficient is defined as follows [41]:

(7) $$\begin{array}{c} d_{ij}^{2}=1-S_{ij}\\ S_{ij}=\frac{\sum_{h=1}^{p1}\left(1-\left|x_{ih}-x_{jh}\right|/G_{h}\right)+a+\alpha }{p1+p2-d+p3}, \end{array}$$

where *p*1 is the number of quantitative variables, *p*2 is the number of binary variables, *p*3 is the number of qualitative variables, *α* is the number of coincidences for qualitative variables, *a* is the number of coincidences in 1 (feature presence) for binary variables, *d* is the number of coincidences in 0 (feature absence) for binary variables, and *G*_*h*_ is the range of the *h*th quantitative variable.

Gower's coefficient is within the range 0-1. A value near to 1 indicates strong similarity between items and a value near to 0 indicates weak similarity.

### 2.5. Metrics to Evaluate the Quality of a Clustering Process

The quality of a clustering process can be evaluated using two types of metrics: internal and external. Internal metrics evaluate the quality of a clustering process based on some intrinsic characteristics, regularly, intra- and intercluster distances. Internal metrics assign high scores to clusters with largest distances among them (separability) and shortest distances among members of the same cluster (compactness). These metrics are very useful when the number of clusters is not known at all. Examples of internal metrics are Q-modularity [42], Davies-Bouldin index, Dunn index, and silhouette [43].

External metrics evaluate the quality of clusters based on data not used during the clustering process, such as the ground truth, that is, the real classes of the instances. The larger the number of instances correctly located according to the ground truth, the higher the index. Some examples of external metrics are Rand index, Folkes and Mallows index, Hubert's T statistic [30], and purity [44].

#### 2.5.1. Purity

The dataset used in this work provides the ground truth. We know there are four classes in the dataset. The objective of this study was to find the features that identify with the highest accuracy possible four clusters, each corresponding to one class. To achieve this goal we selected purity as the metric to evaluate the quality of the clustering process.

Purity validates the quality of a clustering process based on the locations of data in each cluster with respect to the true classes. The more objects in each resultant cluster belong to the true class, the higher the purity. Formally [44],

(8) $$\begin{array}{c} \text{purity}\left(C,W\right)=\frac{1}{N}\underset{k}{\sum }\max j\left(n_{j}^{i}\right), \end{array}$$

where *N* is the number of samples, *C* = {*c*_1_, *c*_2_,…, *c*_*k*_} is the set of clusters found by the clustering algorithm, *W* = {*w*_1_, *w*_2_,…, *w*_*k*_} is the set of the classes of the objects, *n*_*j*_^*i*^ = |*w*_*i*_∩*c*_*j*_| is the number of objects of cluster *i* being in class *j*, *w*_*i*_ is the set of objects in class *k*, and *c*_*j*_ is the set of objects in cluster *k*.

The value of purity ranges from 0 to 1. A purity value of 1 indicates that all the objects in each cluster belong to the same class. An example of purity calculation is shown in Table 1.

The number of objects of the majority class in each cluster is shown in bold. The purity of the clustering is computed as follows: (9 + 14 + 21)/51 = 44/51 = 0.8627.

## 3. Results and Discussion

### 3.1. Experimental Design

We used the 156-feature GBS dataset, described earlier, for experiments. This dataset contains a combination of categorical and numeric features. Gower's coefficient is able to deal with both types of features when present in the same dataset. We used this method to compute the distance matrix among instances, which is required as input to the PAM algorithm.

As we know beforehand, there are four GBS subtypes present in our dataset. This is why the number of clusters requested to PAM algorithm in our experiments was *k* = 4. We expected the clustering algorithm would identify each subtype as a cluster, with the highest purity possible. Five filter methods were used for feature selection, as clustering algorithms perform more efficiently when they work only with relevant attributes [13].

The class attribute was not used when the clustering algorithm was executed. We used it to compute the purity of the clusters obtained with PAM.

A baseline purity using all the 156 features included in the dataset was computed. This value was compared with the purity obtained using only the relevant features as determined by each filter method. Such comparison would allow for a clear view of the benefits of the feature selection process over using the entire dataset, in terms of purity.

Each of the five filter methods selected for experiments in this work was applied to the 156-feature dataset. Along with the features, the class attribute was included in the dataset during the filtering process.

As previously described, CFS and consistency methods include in their output the subset with the most relevant features found. In contrast, chi-squared, information gain, and symmetrical uncertainty methods output a feature ranking.

In all scenarios, new datasets were created with the best feature subsets. The distance matrix of the new datasets was calculated and used as input to the PAM algorithm. Finally, purity of clusters was computed.

In both CFS and consistency methods, the new datasets were created with the resultant most relevant features.

For chi-squared, information gain, and symmetrical uncertainty, feature rankings they produced were used to create the new datasets. Datasets with dimension from 2 through 156 were created, with the best two features, the best three features, and so on. The reason for a dataset of dimension 2 is that the calculation of the distance matrix requires at least 2 attributes. The best feature subset was the set of features conforming the dataset which led to the highest purity in the clustering process. 

### 3.2. Results

#### 3.2.1. Identification of the Four GBS Subtypes

The baseline purity of the four clusters obtained using all the 156 features included in the dataset was 0.6899. After experimentation, four filter methods found feature subsets which increased the baseline purity after the clustering process. Only the feature subset selected by the consistency method as the most relevant obtained a lower purity of 0.6589 than that of the baseline experiment.

Table 2 shows the results of purity of the five methods. Three methods tied with the highest purity (0.7984): information gain, symmetrical uncertainty, and CFS. Both information gain and symmetrical uncertainty selected seven relevant features while CFS selected 16 relevant features. Chi-squared method chose 41 nerve conduction test variables as the most relevant and reached 0.7829 of purity. The consistency method showed the worst performance, which reached a purity of 0.6589. The six relevant features selected by consistency method were two clinical and four corresponding to the nerve conduction test.

Table 3 shows the list of the variables selected by both information gain and symmetrical uncertainty. These variables conformed as a dataset were able to identify the four subtypes of GBS with a purity of 0.7984. All these variables are related to the nerve conduction test.

CFS picked out 16 relevant variables, three of them clinical and 13 corresponding to the nerve conduction test, which reached a purity of 0.7984 as well. The list of 16 variables is shown in Table 4.

Four variables from Tables 3 and 4, denoted by (*), were selected by all methods.

**Figure 1:**
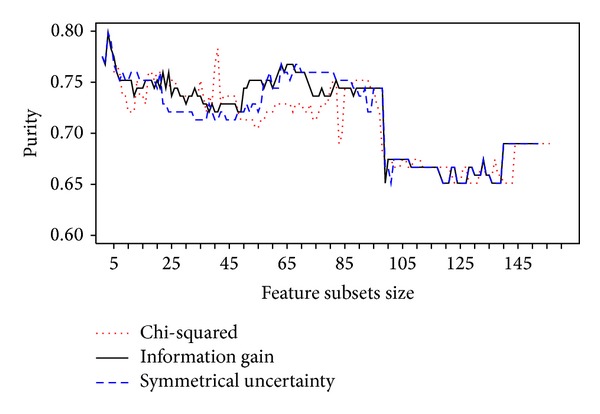
Purity reached by the best feature subsets as ranked by chi-squared, information gain, and symmetrical uncertainty methods.

Purity results of the clustering process using the datasets formed with the most relevant features as ranked by chi-squared, information gain, and symmetrical uncertainty, as described in methodology section, are shown in Figure 1. The three methods behave similarly. Both information gain and symmetrical uncertainty methods reached a maximum value with seven relevant variables, while chi-squared method reached its maximum with 41 variables. All three methods kept purity in the range of 0.7 and 0.8 for feature subsets of sizes between 2 and 102. For bigger subsets, purity lies in the range of 0.65 through 0.7.

#### 3.2.2. Pairwise Exploration of the GBS Subtypes

In order to investigate if any two pairs of GBS subtypes were distinguishable we conducted an additional experiment. We created six new datasets, each one containing instances of only two GBS subtypes. We calculated a baseline purity of each pair of GBS subtypes using all the 156 features. Our goal was to determine a feature subset capable of identifying each pair of GBS subtypes with a higher purity than that of the baseline. We used the five filter methods investigated all along this work to determine the most relevant features for each pair of GBS subtypes. For all scenarios we used *k* = 2, as there are only two GBS subtypes in each dataset. Finally, we applied PAM to form the clusters using only the relevant features determined with each filter method and calculated their purity.

Table 5 shows the results of this experiment. Each row represents a pair of GBS subtypes. Columns 2 to 6 represent a filter method. The right-most column indicates the purity achieved using all the features in the dataset, that is, doing no feature selection at all. Table entries indicate the purity obtained in each case. Numbers in bold show the highest purity obtained for each pair of GBS subtype. Based on the purity obtained, it was found that any filter method is better than using all the features. The highest purity for all pairs of GBS subtypes was superior to 0.9. This result demonstrates the effectiveness of filter methods and highlights the importance of feature selection.

#### 3.2.3. Exploring Different Values of *k*

As explained at the beginning of Section 3.1, we performed the clustering process requesting *k* = 4 clusters as we know this is the number of existing GBS subtypes in the dataset. However, we wanted to explore the clustering process with different values of *k*. Purity results were analyzed and shown in Table 6.

The results of this experiment are shown in Table 6. The first column represents the different values of *k* analyzed. Each remaining column represents a filter method. The right-most column represents the purity obtained using all the features, that is, doing no feature selection at all. Each row represents the results obtained for each value of *k*. Table entries indicate the purity obtained in each case. The results indicate that, in general, purity keeps an ascending pattern as *k* increases. Purities for *k* = 4 and *k* = 5 are very close. In all cases, purity is low for *k* = 2 and very high for *k* = 20. The highest purity values were found for *k* = 20 in all cases; however, these numbers do not indicate that the real number of clusters in the dataset is 20; in fact this number of clusters does not correspond with the nature of GBS subtypes in real life. This result confirms what is reported in literature; higher values of purity are easily obtained for higher values of *k* [45]. Purity is a good evaluation metric for clustering when the number of clusters is known, as in this case.

### 3.3. Discussion

Our objective in this work was to find the best feature subset to identify four GBS subtypes with the highest purity. We did not find any similar work in the literature; therefore this one represents the first effort in this direction. In order to achieve our purpose, we applied machine learning techniques. We used five filter methods for feature selection and compared their performance.

#### 3.3.1. Importance of Feature Selection to Identify GBS Subtypes

The clustering of the four GBS subtypes using all the 156 features in the dataset reached a purity of 0.6899. This means that many cases were mislocated in the clustering process. Table 2 shows that four of the five feature selection methods used in this work obtained a small feature subset that led to the identification of the four groups with a higher purity than that of the baseline.

The identification of GBS subtypes pairwise was achieved with a high purity. The initial baseline purity was improved in all cases (Table 5) when the algorithm used only the relevant features.

These results demonstrate that the clustering algorithm underperforms in the presence of redundant and irrelevant features and highlight the importance of feature selection methods.

#### 3.3.2. Analysis of Different Numbers of Clusters

Purity is a good evaluation metric for clustering when the number of clusters is known, as in this case. Higher purity is easily achieved as the number of clusters increases [45] and that is demonstrated with the results shown in Table 6.

#### 3.3.3. Identification of Four GBS Subtypes

The main contribution of this work is the identification of a subset of seven relevant features from a dataset of 156 variables which identified four GBS subtypes with a purity of 0.7984. Another contribution is the analysis of the performance of five filter methods for feature selection. Finally, this work contributes with the feature rankings produced by chi-squared, information gain, and symmetrical uncertainty methods.

A remarkable finding is that all five methods coincided in four variables. It is also noteworthy that only two of the five methods selected clinical variables. It is important to highlight the fact that the consistency method was not able to select a feature subset to improve the baseline purity (0.6899), but instead the six features selected by this method achieved a worse purity (0.6589).

Information gain, symmetrical uncertainty, and CFS were showed to be highly efficient as they could obtain a reduced subset of relevant features that allow identifying four subtypes of GBS with high purity (0.7984). The first two methods coincided in the same seven variables. CSF selected 16 variables. Further studies are needed to evaluate other methods of feature selection, such as wrapper, embedded, and hybrid methods.

## 4. Conclusions

In this work, we aimed to find a reduced feature subset for identifying four subtypes of GBS with the highest purity. This work represents the first effort on using cluster analysis to identify GBS subtypes. We used for experiments a real dataset of 156 features containing clinical, serological, and nerve conduction tests data. A clustering process was performed with PAM algorithm. In order to select the most relevant features from the dataset as input for PAM, we conducted experiments with five filter methods: CFS, chi-squared, information gain, symmetrical uncertainty, and consistency.

We succeeded as two filter methods were able to find a feature subset consisting of only seven variables that allowed us to obtain a purity of 0.7984. This result originated the first computational characterization of GBS subtypes. Besides, the reduced number of features found to identify the four GBS subtypes could guide physicians to design a faster, simpler, and cheaper diagnosis of the syndrome case.

Other filter methods like FCBF (Fast Correlation-Based Filter) [46] and INTERACT [47] could be used in further studies. Also, more sophisticated methods of feature selection are recommended for analysis, such as those listed in [48–50].

Finally, machine learning techniques such as neural networks or support vector machines could be used for clustering. Purity on their resultant clusters can be compared to that of PAM. This study is planned to further our research.

## Figures and Tables

**Algorithm 1:**
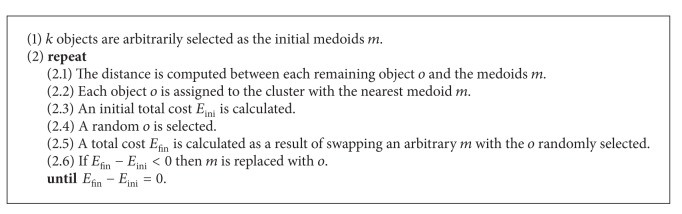
Partitions around medoids (PAM).

**Table 1:** Purity of a clustering with three classes.

	Class A	Class B	Class C	
Cluster 1	0	**14**	1	15
Cluster 2	**9**	2	0	11
Cluster 3	3	1	**21**	25

	12	17	22	**51**

**Table 2:** Results of filter methods ranked on purity.

Method	Number of features	Purity
Information gain	7	0.7984
Symmetrical uncertainty	7	0.7984
CFS	16	0.7984
Chi-squared	41	0.7829
Consistency	6	0.6589

**Table 3:** List of variables with the highest purity (0.7984) selected by information gain and symmetrical uncertainty.

Feature	Meaning
v105	Amplitude of left ulnar motor nerve
v106∗	Area under the curve of left ulnar motor nerve
v116	Amplitude of right ulnar motor nerve
v172∗	Amplitude of left median sensory nerve
v177∗	Amplitude of right median sensory nerve
v182∗	Amplitude of left ulnar sensory nerve
v187	Amplitude of right ulnar sensory nerve

**Table 4:** List of variables with the highest purity (0.7984) selected by CFS.

Feature	Meaning
v29	Extraocular muscles involvement
v30	Ptosis
v40	Karnofsky at discharge
v105	Distal amplitude of left ulnar motor nerve
v106∗	Area under the curve of left ulnar motor nerve
v108	Proximal amplitude of left ulnar motor nerve
v111	Average F-wave latency of left ulnar motor nerve
v116	Distal amplitude of right ulnar motor nerve
v134	F-wave amplitude of left tibial motor nerve
v172∗	Amplitude of left median sensory nerve
v173	Area under the curve of left median sensory nerve
v177∗	Amplitude of right median sensory nerve
v182∗	Amplitude of left ulnar sensory nerve
v185	Conduction velocity of right ulnar sensory nerve
v187	Amplitude of right ulnar sensory nerve
v192	Amplitude of left sural sensory nerve

**Table 5:** Purity of pairwise clustering of GBS subtypes.

GBS subtypes	IG	SU	CFS	Consistency	Chi-squared	All features
AIDP and AMAN	0.9649 (2)	0.9649 (2)	∗	**0.9824 (2)**	0.9649 (2)	0.8771
AMAN and AMSAN	0.927 (3)	0.9375 (2)	0.875 (7)	0.927 (3)	**0.9687 (4)**	0.7395
AIDP and AMSAN	**0.962 (3)**	0.9367 (2)	0.9113 (9)	0.7468 (5)	**0.962 (3)**	0.8354
AIDP and MF	0.8787 (3)	0.8787 (4)	0.8787 (4)	0.8787 (3)	**0.909 (3)**	0.6666
AMAN and MF	**0.98 (6)**	0.96 (3)	**0.98 (4)**	0.74 (2)	**0.98 (6)**	0.96
AMSAN and MF	0.9305 (13)	0.9444 (13)	**0.9583 (14)**	0.8194 (5)	0.9444 (14)	0.8333

IG: information gain, SU: symmetrical uncertainty, and ∗one feature selected therefore purity was not computed. The number of features selected in each case is shown in parenthesis.

**Table 6:** Purity for different numbers of clusters using the four GBS subtypes.

*k*	IG	SU	CFS	Consistency	Chi-squared	All features
2	0.6434 (9)	0.6434 (10)	0.6511 (16)	0.4883 (6)	0.6124 (48)	0.5038
3	0.7906 (7)	0.7906 (7)	0.7286 (16)	0.6666 (6)	0.7829 (6)	0.5813
4	0.7984 (7)	0.7984 (7)	0.7984 (16)	0.6589 (6)	0.7829 (41)	0.6899
5	0.7984 (5)	0.7984 (5)	0.7906 (16)	0.7286 (6)	0.7829 (91)	0.6821
6	0.7751 (4)	0.7751 (7)	0.8139 (16)	0.7596 (6)	0.7751 (38)	0.6666
10	0.8139 (5)	0.8139 (5)	0.8217 (16)	0.7596 (6)	0.8062 (38)	0.6976
20	0.8449 (38)	0.8372 (31)	0.8527 (16)	0.8294 (6)	0.8294 (53)	0.7596

*k*: number of clusters, IG: information gain, and SU: symmetrical uncertainty. The number of features selected in each case is shown in parenthesis.
